# Social media interventions for precision public health: promises and risks

**DOI:** 10.1038/s41746-018-0054-0

**Published:** 2018-09-19

**Authors:** Adam G. Dunn, Kenneth D. Mandl, Enrico Coiera

**Affiliations:** 1 Centre for Health Informatics, Australian Institute of Health Innovation, Macquarie University, Sydney, NSW 2109 Australia; 2 Computational Health Informatics Program, Boston Children’s Hospital, Boston, MA 02115 United States; 3 Department of Biomedical Informatics, Harvard Medical School, Boston, MA 02115 United States; 4 Department of Pediatrics, Harvard Medical School, Boston, MA 02115 United States

**Keywords:** Preventive medicine, Information technology, Risk factors

## Abstract

Social media data can be used with digital phenotyping tools to profile the attitudes, behaviours, and health outcomes of people. While there are a growing number of examples demonstrating the performance of digital phenotyping tools using social media data, little is known about their capacity to support the delivery of targeted and personalised behaviour change interventions to improve health. Similar tools are already used in marketing and politics, using individual profiling to manipulate purchasing and voting behaviours. The coupling of digital phenotyping tools and behaviour change interventions may play a more positive role in preventive medicine to improve health behaviours, but potential risks and unintended consequences may come from embedding behavioural interventions in social spaces.

## Introduction

In 2013, a series of new methods were published demonstrating the possibility of using Facebook ‘likes’ to predict aspects of personality and demographics.^1–3^ These experiments showed the ease with which individuals can be profiled using the digital traces they leave behind online, and generated interest among academics as well as commercial and political organisations. Then in 2017, we saw experimental evidence that these tools can be wielded for the purposes of social manipulation,^4^ as well as evidence that tools based on these methods were being deployed at unprecedented scales to manipulate voting in elections.^5^ News about the way Cambridge Analytica accessed and used Facebook data remind us not only that our personal data can be leveraged to influence our behaviour, but also that the regulatory and ethical frameworks around those activities are underdeveloped.

To understand the role that similar approaches might play in preventive medicine, we examine studies in which social media data are used to predict or model health-related behaviours and outcomes. We then explore how these methods might be operationalised in the design of precision behavioural interventions, and how the effects of these interventions might be amplified or lead to unintended consequences when delivered in a networked public.

## From characterising populations to individual profiling

Changes in the way people live and communicate have made it possible to access data about when people sleep,^6^ when and where they exercise,^7,8^ and track the information they engage with online.^9^ Researchers have used these data in two ways: aggregated to identify signals of population-level outcomes, and at the individual level to predict personal attributes from linked data. Both forms rely on robust measures of behaviours, attitudes, or health outcomes, but the ways they are operationalised to change health behaviours are different.

Population-level studies that aggregate publicly-accessible data have demonstrated the capacity to model spatial variations in voting behaviours,^10^ cardiovascular mortality,^11^ and vaccine coverage.^12^ Studies tend to use Twitter when larger volumes of data are required.^13^ These types of studies are validated against traditional data sources including surveys, disease notifications, and census data. In most cases, data from social media platforms produce a biased representation of location or demography.^14,15^ Accounting for biases in data are important in studies that conclude about incidence and prevalence without validating models against other data sources (especially social media studies that draw conclusions based on number of tweets).^16^ Because studies examining associations between what can be observed on social media and health outcomes have so far been limited to high-prevalence conditions and behaviours like cardiovascular mortality and vaccine coverage,^11,12^ it is not yet clear whether social media data can be used to reliably model rarer outcomes. Population-level studies can be operationalised to complement traditional public health surveillance with faster and less costly information; but tend to produce shallow information and are blunt instruments for designing communication interventions.

Individual-level studies that predict attitudes, behaviours, and health outcomes of people work differently, linking social media user data to validated survey instruments or health records, often using much smaller cohorts. An early example demonstrated the ability to predict major depressive episodes from Twitter data and used validated survey tools to establish diagnoses.^17^ Mental health has become a common topic of focus,^18,19^ though other attitudes, behaviours, and health outcomes have been studied in similar ways.^2,20^ There are no barriers to extending these studies to other phenotypes.^21^ While this approach can work with much smaller cohorts than population-level studies, their construction and validation rely on the quality of the instruments used to measure attitudes, behaviours, and health outcomes of the participants. We expect that this approach will work across major social media platforms and make it possible to detect reasonable signals of suicidal ideation, the misuse of prescription drugs, problem gambling, unhealthy diets, vaccine hesitancy and refusal, and lifestyle factors associated with increased risks of cancer and cardiovascular disease.

## Delivering interventions within a networked public

Effective behaviour change interventions influence the attitudes people hold and the choices they make about their health or the health of their community. Traditional approaches might see a government or public health organisation address problems of vaccine coverage by conducting a survey on vaccine hesitancy to guide the design of a communication intervention; or use population level data about healthcare services to allocate more resources to locations with poorer access. Social media presents an unusual opportunity to identify and deliver personalised digital interventions in an integrated way,^22,23^ and there is evidence that this form of personalised social manipulation can be effective.^4^ When undertaken with individual consent from participants, such behaviour change approaches will live or die based on the merits of their effectiveness. It is a very different question to contemplate deploying such online personalised interventions at scale, without consent, and where targets of the intervention are unaware that they are being manipulated.

The challenges associated with delivering and evaluating population-level digital behaviour change interventions come from the networked nature of online social spaces. Borrowing from Tufekci,^24^ a networked public refers to the complex interactions of people within a society, using communication technologies that facilitate the formation of communities (as structure), as well as the spread of information through those communities (as dynamics). When designing communication interventions to work in social spaces where people are concurrently consumers and broadcasters of information, interactions in the network may be potential confounders or part of the intervention.^22^

The first challenge is evaluation—we are only starting to grapple with the experimental designs needed to test such interventions in trials and in natural settings. Observational evidence shows that health behaviours can be partially explained by the health behaviours and outcomes of families and friends measured in egocentric social networks, including for behaviours related to obesity, smoking, and happiness.^25–27^ Trials that can separate and control for the effects of social network structures are still relatively rare.^28^ Early evidence from studies that insert software agents into artificially-constrained social network structures demonstrate the potential to drive collective behaviour change,^29^ though recent work suggests the form of experiments that may test effectiveness in natural settings.^30^

The second challenge is implementation—social networks may supress or amplify the effects of behaviour change interventions in unpredictable ways. Interventions in this space must compete for attention in an information-rich environment where misinformation may spread faster.^31^ Experiments in agent-based simulations and observational data from social media show that even where individuals have the capacity to discern between high and low quality information, an increased volume of information leads to an increased likelihood that low quality information will spread and persist.^32^ Online social spaces may also amplify the effects of behaviour change interventions. For example, trials testing the effects of messaging interventions aimed at influencing vaccination attitudes often fail to show an effect on behaviour,^33,34^ but this may be because they are tested on individuals in artificial environments rather than in the social spaces where information credibility and beliefs are socially constructed.^35,36^

## Risks and unintended consequences

Backlash is a possible short-term consequence of the use of automated behaviour change tools. The public reaction to an interventional study where Facebook manipulated what users saw to determine its effects on mood was emblematic of what can happen when users discover that they have little control over the information they consume.^37^ Increased use of these methods may represent an erosion of privacy and with it, a perceived threat to individual autonomy.

Medium term consequences might include driving unhealthy behaviours underground. Marlinspike,^38^ in 2013, described how the perceived erosion of privacy that comes with public knowledge of expanded surveillance can create a chilling effect on behaviours, and the importance of privacy even for those who believe they have nothing to hide. Social media users routinely describe using pain drugs, stimulants, and alcohol online. When users discover that organisations are monitoring and manipulating their behaviour, they may adapt by obfuscating what they say or how they interact to avoid being targeted (e.g. when hate speech was targeted on Twitter, users started to use coded language). This would make social media a less reliable signal of behaviour.

Longer term risks may occur if the development of social manipulation methods outpaces the development of countermeasures, where users find ways to hide distinguishing features or limit what they share in the public domain. While there are legitimate reasons for developing and deploying automated behaviour change interventions on social media to improve health, new research efforts in the area could be adapted for use in commercial or political applications. This includes organisations unconstrained by the ethical standards required within academic environments. For example, Cambridge Analytica is suspected to have adapted research from social psychology in an attempt to manipulate voting patterns.^5^

## Research barriers and opportunities

Given the capacity to scale precision behaviour change interventions to societal levels, clear governance structures are now needed to allow for their safe and ethical use. Within academia, ethics reviews will need to consider not only transparency and alignment with participant values but also the broader impact that reporting may have on society. The 2014 experiment in which Facebook modified timelines was an example where users gave consent by agreeing to the terms and conditions of use of the website but the balance of risks versus benefits of changing their timelines to manipulate their emotions may not have warranted.^37,39^ Facebook is not alone. Large internet companies are known for continuously running large numbers of experiments (called A/B tests) without explicitly informing participants. But we typically do now view their impact in the same way because behaviours they seek to change are typically clicks and conversions rather than behaviours that may have direct health implications.

The immediate opportunity in the area comes from linking social media data to surveys and medical records, turning small but high-quality datasets into tools for predicting which individuals are most at risk of certain behaviours or outcomes at societal scales. Methods for iteratively refining predictive models to better target Facebook users are available,^40^ and these are likely to improve identification further. The key difference is that social media also permits direct communication with people that have been traditionally hard to reach,^41,42^ and to reach them well before they visit a clinic or hospital.

## Conclusions

Questions remain about when it is appropriate to couple tools for digital phenotyping with targeted communication interventions to influence health behaviours. There is evidence that digital phenotyping tools have already been weaponised for political propaganda—we have gone from dropping pamphlets from planes to delivering tailored messages directly into the devices that dominate our attention. While the research area is still in its infancy, examples from outside published research leave little doubt that we can take advantage of social media to deliver fully automated, targeted, and cost-effective behaviour change interventions at scale. Despite the volume of health-related social media research published, only a handful of studies have demonstrably predicted health behaviours and outcomes for individuals. While there is a clear potential for their use in improving health outcomes, there are also risks that the adoption of new tools in the area may lead to a perceived threat to autonomy and backlash. Until researchers have the capacity to evaluate them in well-designed studies demonstrating that the benefits outweigh the risks, we recommend caution in their deployment in preventive medicine and public health.
